# Adsorption-mediated modulation of lipopolysaccharide bioactivity by clinoptilolite zeolite with *in vitro* immunomodulatory effects and *in vivo* safety evaluation

**DOI:** 10.3389/fimmu.2026.1818740

**Published:** 2026-07-30

**Authors:** Povilas Kavaliauskas, Rolandas Stankevicius, Julius Rakickas, Alius Pockevicius, Jonas Simkevicius, Ramune Grigaleviciute

**Affiliations:** 1 Department of Animal Nutrition, Lithuanian University of Health Sciences, Kaunas, Lithuania; 2 Biological Research Center, Lithuanian University of Health Sciences, Kaunas, Lithuania; 3 Institute of Infectious Diseases and Pathogenic Microbiology, Prienai, Lithuania; 4 Public Institution Šilutė Primary Health Care Center, Šilutė, Lithuania; 5 Department of Veterinary Pathobiology, Lithuanian University of Health Sciences, Kaunas, Lithuania; 6 UAB Nutrex, Vilnius, Lithuania

**Keywords:** intestinal inflammation, LPS, NF-κB, toll like receptors (TLR), zeolite (clinoptilolite)

## Abstract

**Background:**

Endotoxin-driven intestinal inflammation represents a major contributor to impaired barrier function, metabolic inefficiency, and immune dysregulation in both human and animal health. Strategies that reduce luminal inflammatory triggers rather than directly suppress host signaling pathways represent an emerging paradigm in functional nutrition. Clinoptilolite zeolite, a naturally occurring aluminosilicate mineral with high adsorption capacity, is widely used as a feed additive; however, its capacity to modulate endotoxin-mediated inflammatory signaling has not been mechanistically defined.

**Methods:**

We systematically evaluated the immunomodulatory potential of feed-grade clinoptilolite *in vitro* and assessed its subchronic safety *in vivo*. LPS-induced NF-κB activation was quantified in THP-1 Dual reporter cells, nitric oxide production was measured in RAW264.7 macrophages, and pro-inflammatory cytokine secretion (IL-1β, IL-6, IL-8, and TNF-α) was analyzed in Caco-2 intestinal epithelial cells. To determine whether clinoptilolite reduces endotoxin bioactivity, LPS was pre-incubated with the material prior to cellular stimulation. Systemic tolerability was evaluated in Wistar rats receiving daily oral administration (5–500 mg/kg) followed by comprehensive hematological and histopathological assessments.

**Results:**

Clinoptilolite significantly attenuated LPS-induced NF-κB activation and nitric oxide production in innate immune cells without intrinsic pro-inflammatory activity. Pre-incubation experiments demonstrated a marked reduction in residual LPS bioactivity, supporting an adsorption-mediated mechanism. In intestinal epithelial cells, clinoptilolite selectively reduced LPS-induced IL-6 and IL-8 secretion while preserving IL-1β and TNF-α responses, indicating pathway-specific modulation rather than global immune suppression. Subchronic oral administration produced no clinically relevant hematological alterations or treatment-related histopathological changes in major organs.

**Conclusion:**

These findings provide integrated mechanistic and safety evidence that clinoptilolite zeolite attenuates endotoxin-driven inflammatory signaling by reducing luminal bioavailability while maintaining systemic tolerability. The data support its development as a functional nutritional material aimed at modulating intestinal inflammatory responses without directly targeting host intracellular pathways.

## Introduction

Intestinal inflammation represents a critical challenge in both human and animal health, as disruption of epithelial barrier integrity directly affects nutrient absorption, immune homeostasis, and metabolic performance (1–3). In livestock and companion animal production systems, even subclinical intestinal inflammation can impair feed conversion efficiency, reduce growth rates, alter microbial composition, and increase susceptibility to enteric infections (3, 4). At the molecular level, intestinal inflammation is frequently driven by microbe-derived pathogen-associated molecular patterns (PAMPs), particularly lipopolysaccharide (LPS), a component of the outer membrane of Gram-negative bacteria and a microbiota-produced PAMP (5–8). Recognition of LPS by the Toll-like receptor 4 (TLR4)/MD-2/CD14 signaling complex activates downstream NF-κB-dependent inflammatory pathways, resulting in the production of pro-inflammatory cytokines, chemokines, and mediators that contribute to epithelial dysfunction and increased intestinal permeability (8, 9). Persistent activation of these inflammatory cascades promotes disruption of tight junction architecture, facilitates translocation of luminal microbial products, and amplifies local immune activation, ultimately compromising intestinal barrier homeostasis (10). Consequently, strategies capable of reducing LPS-induced inflammatory signaling or preserving epithelial barrier integrity have attracted increasing interest as supportive approaches for improving intestinal health and resilience in both veterinary and biomedical settings (11).

LPS activates Toll-like receptor 4 (TLR4) on epithelial and immune cells, triggering downstream signaling cascades involving MyD88- and TRIF-dependent pathways, culminating in activation of the nuclear factor kappa B (NF-κB) transcription factor (12–14). This signaling axis drives the expression of pro-inflammatory cytokines, including INF-y, TNF-α, IL-6, IL-8, and inducible nitric oxide synthase (iNOS), perpetuating mucosal immune activation and contributing to chronic low-grade inflammation (15, 16). Persistent activation of this pathway compromises epithelial barrier function and exacerbates metabolic inefficiencies in production animals.

Given the global reduction in antibiotic use in animal agriculture and the increasing demand for sustainable feed solutions, there is a pressing need for non-pharmacological strategies capable of modulating endotoxin-driven inflammation at the intestinal interface (17, 18). Rather than directly suppressing immune signaling, an alternative approach involves reducing the luminal bioavailability of inflammatory triggers such as LPS before they engage host receptors (19).

Clinoptilolite zeolites (zeolite) are naturally occurring crystalline aluminosilicate minerals characterized by a highly ordered microporous framework, large surface area, and pronounced cation-exchange capacity (20, 21). These physicochemical properties enable clinoptilolite zeolites to adsorb a broad spectrum of luminal compounds, including ammonia, mycotoxins, heavy metals, and bacterial metabolites (21, 22). In animal nutrition, clinoptilolite zeolites have been widely used as feed conditioners and detoxifying agents. However, despite their established adsorption capacity, their potential role in modulating endotoxin-mediated inflammatory signaling has not been systematically evaluated at the molecular level (23–25).

Beyond simple toxin sequestration, the capacity of clinoptilolite zeolite to reduce endotoxin bioavailability may indirectly attenuate TLR4–NF-κB activation and downstream cytokine production in intestinal epithelial and immune cells (22). This adsorption-based mechanism represents a mechanistically distinct strategy from pharmacological anti-inflammatory agents and aligns with the functional feed additive concept, in which materials modulate luminal triggers rather than directly target host intracellular pathways (20).

In the present study, we provide a systematic *in vitro* and proof-of-concept evaluation of natural clinoptilolite zeolite as a functional material capable of modulating endotoxin-driven inflammatory signaling. We demonstrate that clinoptilolite zeolite attenuates LPS-induced NF-κB activation, nitric oxide production, and pro-inflammatory cytokine secretion in immune and intestinal epithelial cell models. Functional pre-incubation experiments demonstrate reduced endotoxin bioactivity. Importantly, subchronic oral administration in rats confirmed hematological and histological tolerability. Together, these findings position clinoptilolite zeolite as a promising dietary material with the potential to modulate intestinal inflammatory responses while maintaining systemic safety.

## Materials and methods

### Clinoptilolite zeolite material

Natural clinoptilolite zeolite powder (product code 2347, ZeoFeed^®^ B1 BB pal.) was obtained from UAB Nutrex (Lithuania). The material was originally manufactured by ZEOCEM s.r.o. (Bystré, Slovakia). Heavy metal concentrations were below the following limits: Pb ≤30 ppm, Cd ≤2 ppm, As ≤2 ppm, and Hg ≤0.1 ppm. Fluoride content was ≤150 ppm. Dioxins were ≤0.75 ng WHO-PCDD/F-TEQ/kg, and the sum of dioxins and dioxin-like PCBs was ≤1.5 ng WHO-PCDD/F-TEQ/kg, indicating chemical purity consistent with feed-grade safety standards (**Supplementary Table 1**). For tissue culture experiments, the clinoptilolite zeolite was sterilized by autoclaving.

### Cell lines and culture conditions

The THP-1 Dual™ NF-κB reporter cell line (InvivoGen, San Diego, CA, USA) was maintained in RPMI-1640 medium (Gibco, Thermo Fisher Scientific, Waltham, MA, USA) supplemented with 10% heat-inactivated fetal bovine serum (FBS; Sigma-Aldrich, St. Louis, MO, USA), 1% penicillin-streptomycin (PenStrep; Gibco, Thermo Fisher Scientific), and Normocin™ (100 µg/mL; InvivoGen), according to the manufacturer’s instructions. Cells were cultured as a suspension cell line and passaged by replacing the culture medium twice weekly. RAW 264.7 murine macrophages (ATCC^®^ TIB-71™, American Type Culture Collection, Manassas, VA, USA) were cultured in RPMI-1640 medium supplemented with 10% heat-inactivated FBS and 1% penicillin-streptomycin (hereafter referred to as complete RPMI-1640 medium). Cells were maintained as adherent monolayers and passaged twice weekly using trypsinization. Caco-2 human colorectal adenocarcinoma cells (ATCC^®^ HTB-37™, American Type Culture Collection) were cultured in DMEM/F-12 medium supplemented with GlutaMAX™ (Gibco, #10565018, Thermo Fisher Scientific), 10% heat-inactivated FBS (Gibco, #A3160501, Thermo Fisher Scientific), and 1% penicillin-streptomycin (Gibco, #10378016, Thermo Fisher Scientific) (hereafter referred to as complete DMEM/F-12 medium). Cells were maintained as adherent monolayers and subcultured twice weekly. Unless otherwise specified, all cell lines were cultured at 37 °C in a humidified incubator with 5% CO_2_ atmosphere.

### Preparation of clinoptilolite zeolite suspension for tissue culture stimulation experiments

Dry clinoptilolite zeolite powder was sterilized by autoclaving (121 °C, 15 min) and subsequently dried overnight at 40 °C in an incubator to remove residual moisture. Following sterilization, 100 mg of clinoptilolite zeolite powder was transferred to sterile 1.5 mL polypropylene microcentrifuge tubes (Eppendorf, Hamburg, Germany) under aseptic conditions. To remove potential soluble contaminants and loosely associated fractions, the powder was washed three times with the respective tissue culture medium: complete RPMI-1640 for THP-1 Dual and RAW 264.7 cells and complete DMEM/F-12 medium for Caco-2 cells. Each wash consisted of resuspension in sterile medium followed by centrifugation at 10,000 × g for 5 min at room temperature. The supernatant was carefully discarded after each centrifugation step. After the final wash, clinoptilolite zeolite was resuspended in the complete culture medium to obtain a stock suspension of 10 mg/mL. The suspension was mixed thoroughly prior to each use to ensure uniform particle dispersion.

### THP-1 Dual NF-κB reporter cell stimulation with lipopolysaccharide and clinoptilolite zeolite

For stimulation experiments, THP-1 Dual cells were collected by centrifugation (1000 × g, 5 min), washed twice with complete RPMI-1640 medium lacking Normocin™, and resuspended in antibiotic-free complete medium. Cells were seeded into flat-bottom 96-well tissue culture plates (Corning, Corning, NY, USA) at a density of 2 × 10^4^ cells per well in a final volume of 100 µL and allowed to equilibrate overnight under standard culture conditions (37 °C, 5% CO_2_). Sterile clinoptilolite zeolite suspensions were freshly prepared in complete antibiotic-free medium and diluted to a final concentration of 50 µg/mL. To ensure homogeneous dispersion, the suspension was continuously mixed immediately before and during addition to the cells. The clinoptilolite zeolite-containing medium was added to the wells, and cells were pre-incubated for 3 h at 37 °C. Following pre-incubation, lipopolysaccharide (LPS) from *Escherichia coli* O111:B4 (Sigma-Aldrich, St. Louis, MO, USA) was added to each well at a final concentration of 100 ng/mL. Cells were further incubated for 24 h at 37 °C in 5% CO_2_ atmosphere. After stimulation, plates were centrifuged (1,000 × g, 5 min) to pellet cells and particulate material. Supernatants were carefully collected and transferred to a new 96-well plate for quantification of NF-κB activation using the QUANTI-Blue™ reagent (InvivoGen), according to the manufacturer’s instructions. Relative NF-κB activation was expressed as optical density (OD) values and compared with medium-alone or clinoptilolite zeolite control conditions.

### LDH assay for cytotoxicity determination

To quantify treatment-induced cytotoxicity, lactate dehydrogenase (LDH) release was measured using a commercially available assay kit (CyQUANT™ LDH Cytotoxicity Assay, Thermo Fisher Scientific, Waltham, MA, USA), according to the manufacturer’s instructions. Briefly, conditioned media collected from THP-1 Dual, RAW264.7, and Caco-2 cell experiments were transferred to 96-well plates, and LDH activity was quantified following the manufacturer’s protocol.

### Nitric oxide quantification by Griess assay

Nitric oxide production was assessed by measuring nitrite accumulation in culture supernatants using a Griess Reagent Kit (Thermo Fisher Scientific, Waltham, MA, USA), according to the manufacturer’s instructions (26). RAW264.7 murine macrophages were seeded into 12-well tissue culture plates (Corning, Corning, NY, USA) at a density of 5 × 10^5^ cells per well in complete RPMI-1640 medium supplemented with 10% heat-inactivated fetal bovine serum (Gibco, Thermo Fisher Scientific) and 1% penicillin-streptomycin (Gibco). Cells were allowed to adhere overnight at 37 °C in a humidified atmosphere containing 5% CO_2_. The following day, cells were pre-incubated with clinoptilolite zeolite at the same concentration used in the NF-κB reporter experiments (50 µg/mL) for 3 h. Lipopolysaccharide was then added, and cells were further incubated for 6 h under standard culture conditions. After stimulation, culture supernatants were collected and clarified by centrifugation (10,000 × g, 5 min) to remove cellular debris and residual particulate material. Equal volumes of clarified supernatant and Griess reagents were mixed in a flat-bottom 96-well plate and incubated for 10 min at room temperature, protected from light. Absorbance was measured at 540 nm using a microplate reader.

### Lipopolysaccharide binding assay

To evaluate whether clinoptilolite zeolite reduces the availability of LPS to cells in culture medium, lipopolysaccharide (LPS) was diluted in complete RPMI-1640 medium to a final concentration of 100 ng/mL. Clinoptilolite zeolite suspension (prepared as described above) was added to the LPS-containing medium at the indicated concentration and incubated for 3 h at 37 °C under continuous orbital agitation (150 rpm) to facilitate particle–ligand interaction. Control samples containing LPS without clinoptilolite zeolite were processed in parallel. Following incubation, samples were centrifuged at 10,100 × g for 15 min at room temperature to pellet clinoptilolite zeolite particles. The supernatant was carefully collected without disturbing the pellet and filtered through a 0.4 µm syringe filter to eliminate clinoptilolite zeolite particles. The clarified supernatant was immediately used to stimulate THP-1 Dual™ NF-κB reporter cells as described above. Residual LPS activity in the supernatant was assessed indirectly by measuring NF-κB activation in THP-1 Dual cells using the QUANTI-Blue™ assay (InvivoGen), according to the manufacturer’s protocol.

### Caco-2 cell stimulation and cytokine quantification

Caco-2 human colorectal adenocarcinoma cells (ATCC^®^ HTB-37™, American Type Culture Collection, Manassas, VA, USA) were seeded into 6-well tissue culture plates (Corning, Corning, NY, USA) at a density of 5 × 10^5^ cells per well in complete DMEM/F-12 medium. Cells were incubated overnight at 37 °C in a humidified incubator with 5% CO_2_ to allow attachment. The following day, the culture medium was replaced with complete DMEM/F-12 containing clinoptilolite zeolite suspension at a final concentration of 100 µg/mL. Cells were pre-incubated with clinoptilolite zeolite for 3 h at 37 °C. Control wells containing medium alone and clinoptilolite zeolite alone (without LPS stimulation) were included in all experiments. After pre-incubation, lipopolysaccharide was added directly to each well at a final concentration of 1 µg/mL to induce cytokine production. Cells were further incubated for 24 h under standard culture conditions (37 °C, 5% CO_2_). Following stimulation, plates were placed on ice for 10 min. Conditioned media were collected and supplemented with a protease inhibitor cocktail (Sigma, United States) to achieve a final 1× working concentration. Samples were centrifuged at 15,000 × g for 10 min at 4 °C to remove cellular debris and residual particulate material. The clarified supernatant was carefully collected and stored at −80 °C until cytokine quantification by enzyme-linked immunosorbent assay (ELISA), according to the manufacturer’s instructions.

### Cytokine quantification by enzyme-linked immunosorbent assay

Conditioned media samples from Caco-2 stimulation experiments were thawed on ice and briefly mixed prior to analysis. Cytokine concentrations were quantified using commercially available human ELISA kits according to the manufacturers’ instructions. Specifically, interleukin-1β (IL-1β; R&D Systems, #DLB50, R&D Systems, Minneapolis, MN, USA), interleukin-6 (IL-6; Invitrogen, #88-7066-88, Thermo Fisher Scientific, Waltham, MA, USA), interleukin-8 (IL-8; Invitrogen, #KHC0081, Thermo Fisher Scientific), and tumor necrosis factor-α (TNF-α; Human TNF-α Uncoated ELISA Kit, #88-7346-88, Invitrogen, Thermo Fisher Scientific) were measured. For coated ELISA kits, standards and samples were added to pre-coated microplates and incubated according to the manufacturer’s protocol. Standard curves were generated using recombinant cytokine standards provided in each kit. Cytokine concentrations in conditioned media were calculated from standard curves and expressed as pg/mL.

### TLR4 inhibition assay

THP-1 Dual™ NF-κB reporter cells were seeded and cultured under the same conditions described for the NF-κB reporter stimulation assays. To inhibit TLR4 signaling, cells were pre-treated with the small-molecule TLR4 inhibitor TAK-242 (27) (MedChemExpress, HY-11109) at a final concentration of 5 µM for 1 h prior to stimulation. TAK-242 was prepared in dimethyl sulfoxide (DMSO), and vehicle control wells received 0.1% DMSO. Following pre-treatment, cells were stimulated with lipopolysaccharide (LPS) alone or in combination with clinoptilolite zeolite under the same experimental conditions described for the NF-κB activation experiments.

After incubation, conditioned media were collected, and NF-κB reporter activation was quantified using the QUANTI-Blue™ assay (InvivoGen) according to the manufacturer’s instructions. Optical density was measured at 620 nm using a microplate reader.

### FITC-dextran permeability assay

To evaluate epithelial barrier permeability, Caco-2 cells were seeded onto 0.4-µm pore size Transwell inserts (Corning, Corning, NY, USA) at a density of 5 × 10^5^ cells per insert and cultured in complete DMEM/F-12 medium for 7 days under standard culture conditions (37 °C, 5% CO_2_) to allow the formation of epithelial monolayers. Following monolayer establishment, the culture medium was replaced with fresh medium containing either medium alone (untreated control), lipopolysaccharide (LPS), clinoptilolite zeolite (ZEO), or clinoptilolite zeolite together with LPS (ZEO + LPS). Cells were further incubated for 24 h under standard culture conditions.

Following treatment, Transwell inserts were carefully removed and washed three times with Dulbecco’s phosphate-buffered saline (DPBS) to remove residual medium and non-adherent material. Inserts were subsequently transferred to new 6-well plates containing 1 mL of Hank’s Balanced Salt Solution (HBSS) in the basolateral compartment. Fluorescein isothiocyanate (FITC)-dextran (70 kDa; MedChemExpress, HY-128868E) dissolved in HBSS was added to the apical compartment at a final concentration of 250 µg/mL. Plates were incubated for 1 h at 37 °C in a humidified 5% CO_2_ atmosphere to allow FITC-dextran diffusion across the epithelial barrier.

After incubation, Transwell inserts were removed, and HBSS from the basolateral compartment was collected. FITC-dextran fluorescence was quantified using a fluorescence microplate reader at excitation/emission wavelengths (Ex./Em.) of 485/528 nm. Increased fluorescence intensity in the basolateral compartment was interpreted as increased epithelial permeability.

### Ethics of animal studies

Health status and general behavior were monitored daily. Body weight and food intake were recorded at regular intervals during the experimental period. All procedures were conducted in accordance with national and institutional guidelines for the care and use of laboratory animals and complied with the European Directive 2010/63/EU for animal experiments. The animal studies were approved by the State Food and Veterinary Service (SFVS) animal care and welfare committee (approval number: G2-291).

### Experimental animals and housing conditions

Wistar rats were obtained from the Laboratory Animal Center of the Lithuanian University of Health Sciences (Kaunas, Lithuania) and used for the study. At the beginning of the experiment, animals were 8–12 weeks old and weighed 250–400 g. All animals were clinically healthy and acclimatized for at least 7 days prior to experimental procedures. Rats were housed in groups of five per cage in standard polypropylene cages with stainless steel wire lids and autoclaved bedding. Environmental conditions were maintained under controlled parameters: temperature, 21 °C–23 °C; relative humidity, 45%–65%; and a 12 h light/12 h dark cycle. Animals had *ad libitum* access to standard laboratory rodent chow and filtered drinking water throughout the study period.

### Study design and clinoptilolite zeolite administration

A subchronic oral exposure study was conducted to evaluate the safety profile of clinoptilolite zeolite following repeated administration. After acclimatization, animals were randomly assigned to experimental groups (n = 5 per group) receiving clinoptilolite zeolite suspension in water at doses of 5, 50, or 500 mg/kg body weight. The control group received the vehicle only. Clinoptilolite zeolite suspensions were freshly prepared daily in sterile distilled water and administered once daily by oral gavage. The treatment period lasted for 29 consecutive days. Animals were observed daily for signs of clinical toxicity, including changes in behavior, grooming, posture, locomotion, and food and water intake. Body weights were recorded weekly throughout the study. At the end of the treatment period, animals were euthanized using a pentobarbital injection. Blood samples were collected for hematological analysis via cardiac puncture in EDTA-treated syringes.

### Tissue processing and histological analysis

Brain, heart, kidney, spleen, liver, and intestinal tissues were harvested for gross examination and further histopathological evaluation. Tissues were fixed in 10% neutral formaldehyde for 2 days at room temperature. Tissues were then paraffin-embedded using an automated tissue-processing machine. Paraffin-embedded tissues were then sectioned and stained using hematoxylin and eosin (H&E) in an automated staining machine.

### Hematological analysis

Freshly collected whole blood samples were analyzed immediately after collection using an automated hematology analyzer (Vetscan HM5). Blood was collected into tubes containing EDTA anticoagulant and gently inverted prior to analysis. The following hematological parameters were evaluated: total white blood cell count (WBC), lymphocyte percentage, granulocyte percentage, red blood cell count (RBC), hemoglobin concentration (HGB), mean corpuscular volume (MCV), mean corpuscular hemoglobin (MCH), platelet count (PLT), and mean platelet volume (MPV). All analyses were performed in accordance with the manufacturer’s instructions and routine laboratory quality control procedures.

### Statistical analysis

All data were collected and analyzed using GraphPad Prism software (GraphPad Software, San Diego, CA, USA). Data are presented as mean ± standard deviation (SD) unless otherwise indicated. Comparisons between experimental groups and control animals were performed using an unpaired Student’s *t*-test. A two-tailed *p* value < 0.05 was considered statistically significant. For *in vitro* experiments, analyses were conducted using data obtained from three independent experiments. Histological evaluations were performed descriptively. Tissue sections were examined by a pathologist who was blinded to the experimental design and treatment groups. Observations were recorded qualitatively, and no semi-quantitative scoring system was applied.

## Results and discussion

### Clinoptilolite zeolite attenuates LPS-induced NF-κB activation and nitric oxide production in innate immune cell models

**Figure 1:**
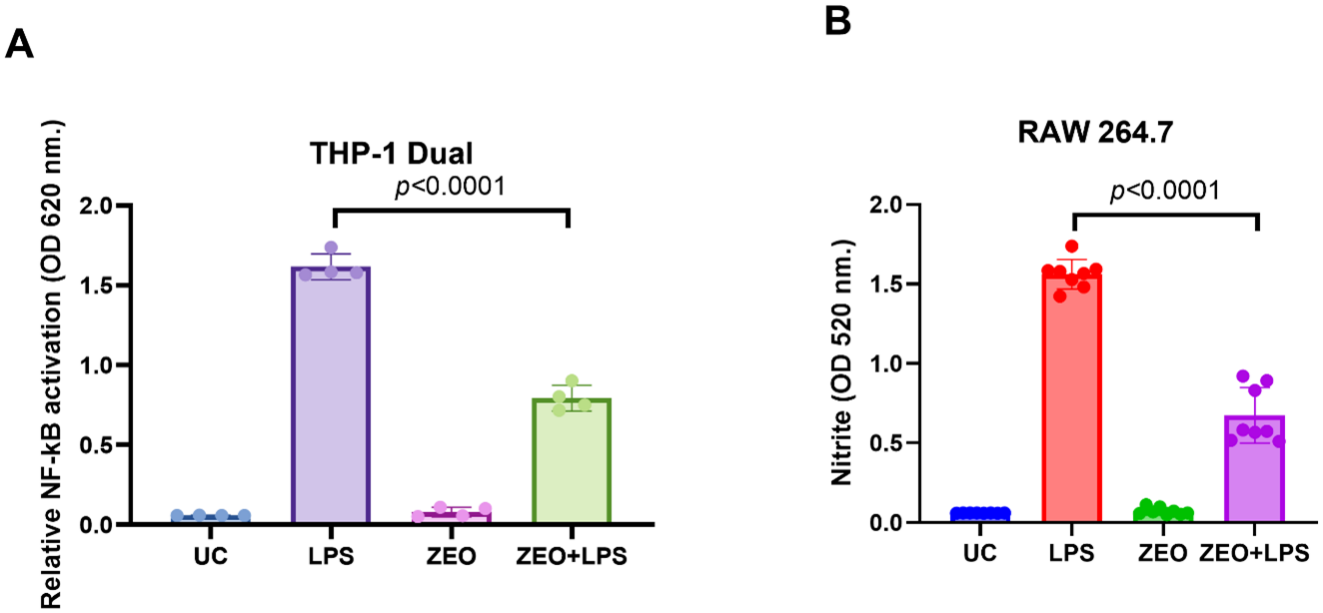
Clinoptilolite zeolite attenuates LPS-induced inflammatory activation in innate immune cells. **(A)** NF-κB activation in THP-1 Dual reporter cells following stimulation with LPS in the presence or absence of clinoptilolite zeolite. **(B)** Nitric oxide production in RAW264.7 macrophages was measured by Griess assay under identical conditions. UC, untreated control; LPS, lipopolysaccharide; ZEO, clinoptilolite zeolite. Data are presented as mean ± SD from three independent experiments.

To understand the immunomodulatory activity of clinoptilolite zeolite, we first evaluated its effect on LPS-induced inflammatory signaling in human THP-1 Dual NF-κB reporter cells and murine RAW264.7 macrophages. Stimulation of THP-1 Dual cells with LPS (100 ng/mL) resulted in a robust increase in NF-κB reporter activity compared with unstimulated controls (UC) (**Figure 1A**).

Clinoptilolite zeolite alone did not induce NF-κB activation, demonstrating that the clinoptilolite zeolite itself does not activate inflammatory signaling. Notably, pre-incubation with clinoptilolite zeolite significantly reduced LPS-induced NF-κB activation *(p* < 0.01) in THP-1 Dual cells, indicating that clinoptilolite zeolite attenuates endotoxin-driven NF-κB activation. Clinoptilolite zeolite alone did not induce substantial cytotoxicity in THP-1 Dual cells, suggesting that the observed phenotype is unlikely to be mediated by treatment-induced cytotoxicity (**Supplementary Figure 1A**). LPS is a potent activator of Toll-like receptor 4 (TLR4), leading to downstream activation of NF-κB and transcription of pro-inflammatory genes. The reduction in NF-κB reporter activity observed in THP-1 cells indicates that clinoptilolite zeolite interferes with early transcriptional activation events.

In order to determine whether clinoptilolite zeolite also affects downstream NF-κB-associated inflammatory responses, we evaluated nitric oxide production in RAW264.7 macrophages by measuring nitrite accumulation in conditioned media using the Griess assay. Similarly, in RAW264.7 macrophages (**Figure 1B**), LPS stimulation markedly increased nitrite accumulation in culture supernatants, reflecting nitric oxide production. Importantly, co-treatment with clinoptilolite zeolite significantly reduced LPS-induced nitrite levels (*p* < 0.0001), suggesting suppression of macrophage inflammatory activation. Consistent with the results observed in THP-1 Dual cells, the marked decrease in nitrite production in RAW264.7 cells suggests attenuation of downstream inflammatory effector responses (28, 29) (**Figure 1B**). Importantly, clinoptilolite zeolite alone did not induce NF-κB activation or nitric oxide production in either cell type, nor did it induce substantial cytotoxicity, as measured by LDH release (**Supplementary Figure 1A, B**), supporting the notion that the material does not intrinsically activate innate immune signaling.

**Figure 2:**
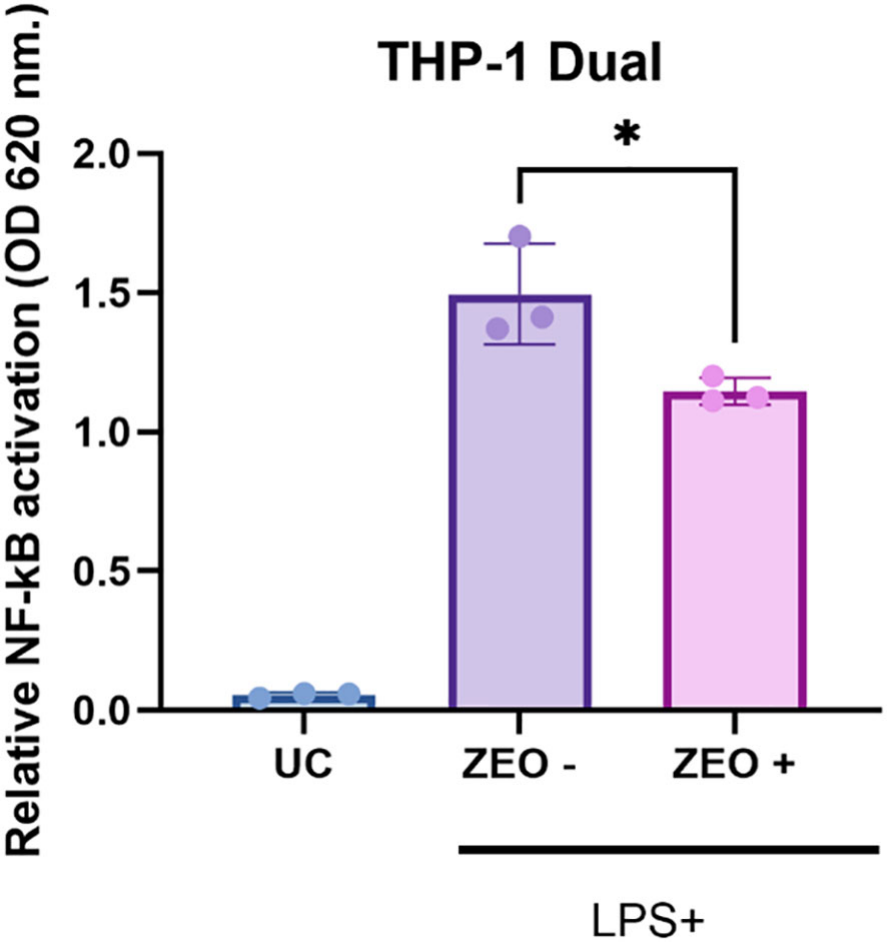
Clinoptilolite zeolite reduces residual LPS bioactivity following pre-incubation in cell-free medium. THP-1 Dual NF-κB reporter cells were stimulated with supernatants obtained after incubation of LPS with clinoptilolite zeolite. NF-κB activation was quantified by reporter assay. Data are presented as mean ± SD. *p < 0.05.

We then aimed to determine whether clinoptilolite zeolite activity is based on direct binding of LPS or modulation of cellular receptors and downstream signaling pathways. To address this question, LPS was pre-incubated with clinoptilolite zeolite in cell-free medium, after which clinoptilolite zeolite particles were removed by high-speed centrifugation and membrane filtration. The clarified supernatant was subsequently used to stimulate THP-1 Dual NF-κB reporter cells (**Figure 2**).

Pre-incubation of LPS with clinoptilolite zeolite in cell-free medium significantly reduced subsequent NF-κB activation in THP-1 Dual reporter cells compared with LPS-containing supernatants incubated in the absence of clinoptilolite zeolite (**Figure 2**). These findings support the interpretation that clinoptilolite zeolite reduces the availability of LPS to stimulate immune cells following pre-incubation in extracellular medium.

Collectively, these results demonstrate that clinoptilolite zeolite significantly attenuates NF-κB activation in THP-1 Dual reporter cells and reduces nitric oxide production in RAW264.7 macrophages following LPS stimulation, possibly by absorbing LPS from tissue culture media.

### Clinoptilolite zeolite-mediated reduction of NF-κB activation requires direct presence during LPS stimulation

**Figure 3:**
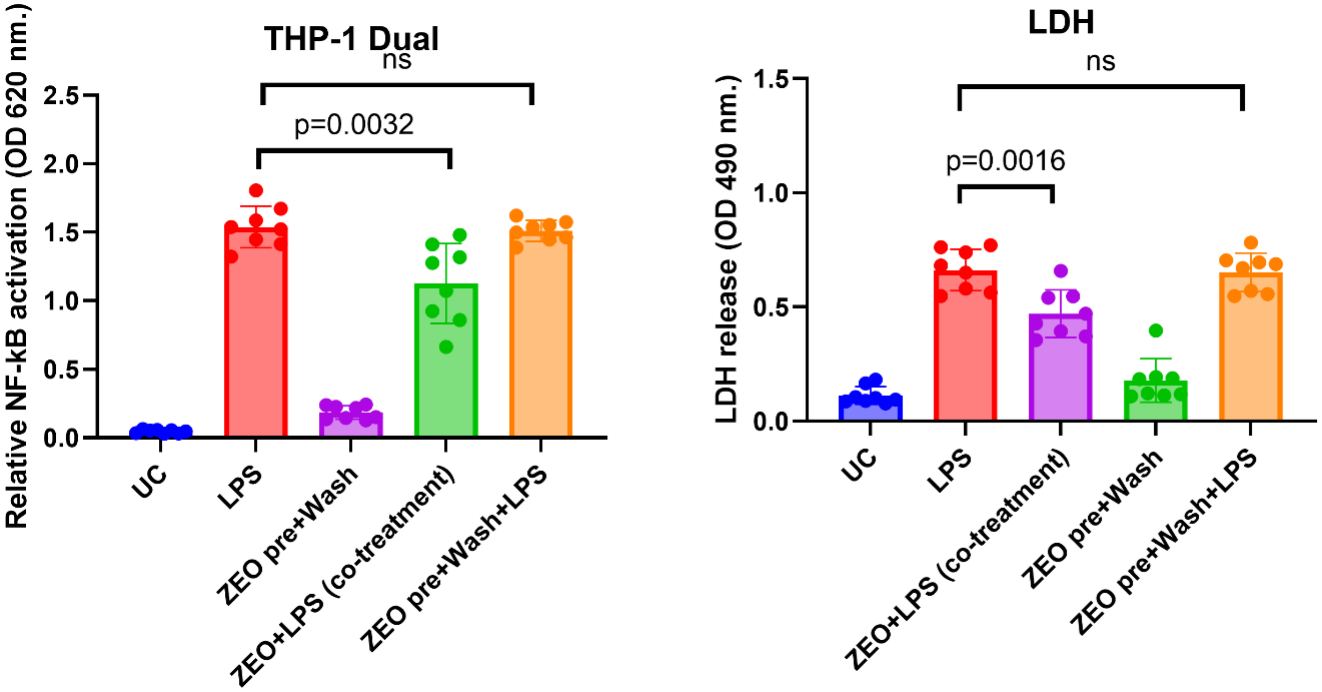
Direct clinoptilolite zeolite exposure during LPS stimulation is required for reduced NF-κB activation in THP-1 Dual cells. THP-1 Dual NF-κB reporter cells were either stimulated with lipopolysaccharide (LPS) in the presence of clinoptilolite zeolite (ZEO + LPS) or pre-exposed to clinoptilolite zeolite placed in 0.4-µm Transwell inserts followed by insert removal, cell washing, and subsequent LPS stimulation (ZEO pre-treatment + LPS). LDH release was measured under the tested conditions to quantify treatment-induced cytotoxicity. Data are presented as mean ± SD. Statistical significance was determined using an unpaired Student’s t-test.

To further investigate whether clinoptilolite zeolite modulates cellular responsiveness independently of extracellular LPS sequestration, THP-1 Dual NF-κB reporter cells were pre-exposed to clinoptilolite zeolite placed in Transwell inserts prior to complete removal of the material and subsequent LPS stimulation (**Figure 3**; **Supplementary Figure 2**). This experimental design was used to distinguish direct extracellular interactions between clinoptilolite zeolite and LPS from potential persistent effects on cellular signaling responsiveness following prior zeolite exposure.

Consistent with previous observations, simultaneous exposure of THP-1 Dual cells to clinoptilolite zeolite and LPS significantly reduced NF-κB activation compared with LPS stimulation alone (*p* = 0.0032) (**Figure 3**). In contrast, pre-treatment of cells with clinoptilolite zeolite followed by insert removal and extensive washing prior to LPS stimulation did not significantly reduce subsequent NF-κB activation compared with LPS-treated controls (**Figure 3**). These findings suggest that the presence of clinoptilolite zeolite during LPS exposure is required for the observed reduction in inflammatory reporter activity.

To exclude the possibility that differential NF-κB responses were secondary to treatment-associated cytotoxicity, LDH release assays were performed under the same experimental conditions (**Figure 3**). No significant increase in LDH release was observed following clinoptilolite zeolite pre-treatment and washing compared with control groups, indicating the absence of substantial membrane-associated cytotoxicity (**Figure 3**).

Collectively, these findings support the interpretation that reduced NF-κB activation primarily reflects an extracellular reduction in LPS availability during direct clinoptilolite zeolite exposure rather than persistent modulation of downstream intracellular inflammatory signaling pathways.

### Zeolite reduces LPS-induced NF-κB activation in a TLR4-dependent manner

**Figure 4:**
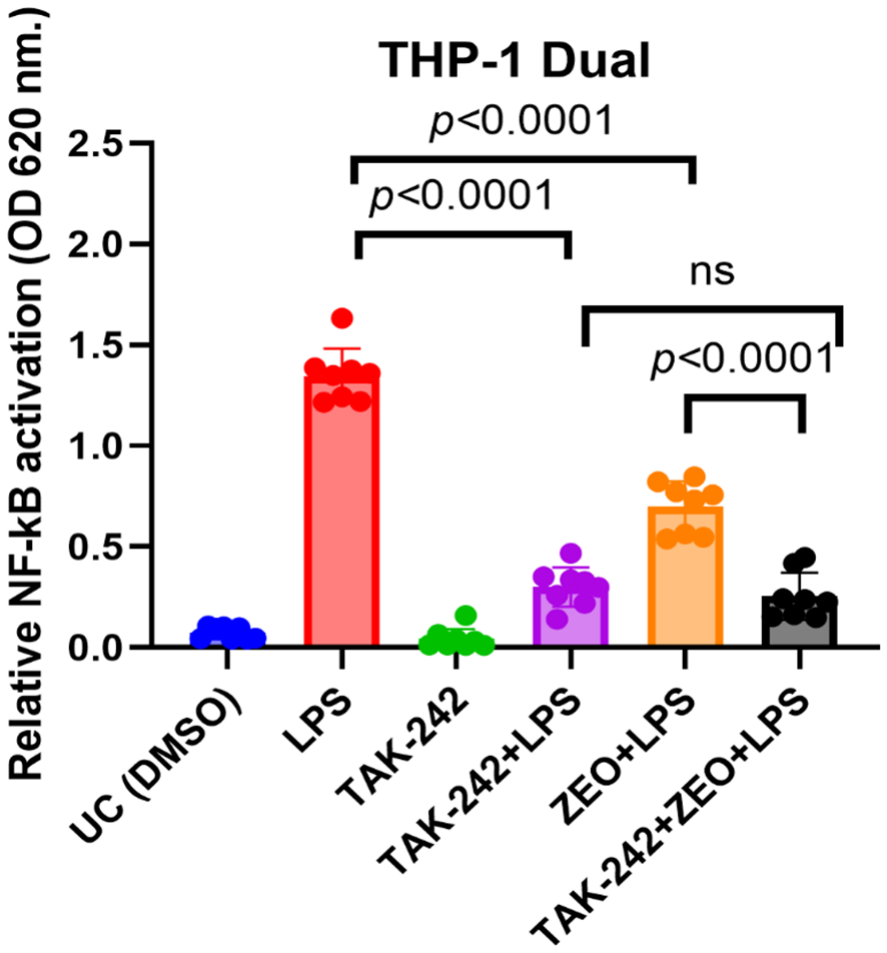
TLR4 inhibition supports zeolite-mediated reduction of LPS-induced NF-κB activation in THP-1 Dual cells. THP-1 Dual NF-κB reporter cells were pre-treated with the small-molecule TLR4 inhibitor TAK-242 (5 µM) for 1 h prior to stimulation with lipopolysaccharide (LPS) or combined clinoptilolite zeolite and LPS treatment conditions. DMSO (0.1%) was included as a vehicle control. Data are presented as mean ± SD. Statistical significance was determined using an unpaired Student’s t-test. ns, not significant.

To further validate that the inflammatory reporter activity modulated by clinoptilolite zeolite was associated with LPS–TLR4 signaling, THP-1 Dual NF-κB reporter cells were treated with the small-molecule TLR4 inhibitor TAK-242 (27, 30, 31) prior to LPS stimulation (**Figure 4**).

As expected, stimulation with LPS alone resulted in robust NF-κB activation compared with untreated controls. Consistent with previous findings, simultaneous exposure of THP-1 Dual cells to clinoptilolite zeolite and LPS significantly reduced NF-κB activation compared with LPS stimulation alone (*p* < 0.0001) (**Figure 4**).

Pre-treatment of THP-1 Dual with TAK-242, a potent TLR4 inhibitor, markedly reduced LPS-induced NF-κB activation, confirming that the reporter signal measured under the experimental conditions employed was predominantly mediated through TLR4-dependent signaling pathways. Importantly, combined treatment with TAK-242 and clinoptilolite zeolite did not further substantially reduce NF-κB activation compared with TAK-242-treated cells stimulated with LPS (**Figure 4**).

These findings support the interpretation that clinoptilolite zeolite reduces inflammatory reporter activation in a system driven by LPS–TLR4 signaling, potentially by limiting LPS availability.

### Clinoptilolite zeolite attenuates LPS-induced cytokine production in Caco-2 intestinal epithelial cells

**Figure 5:**
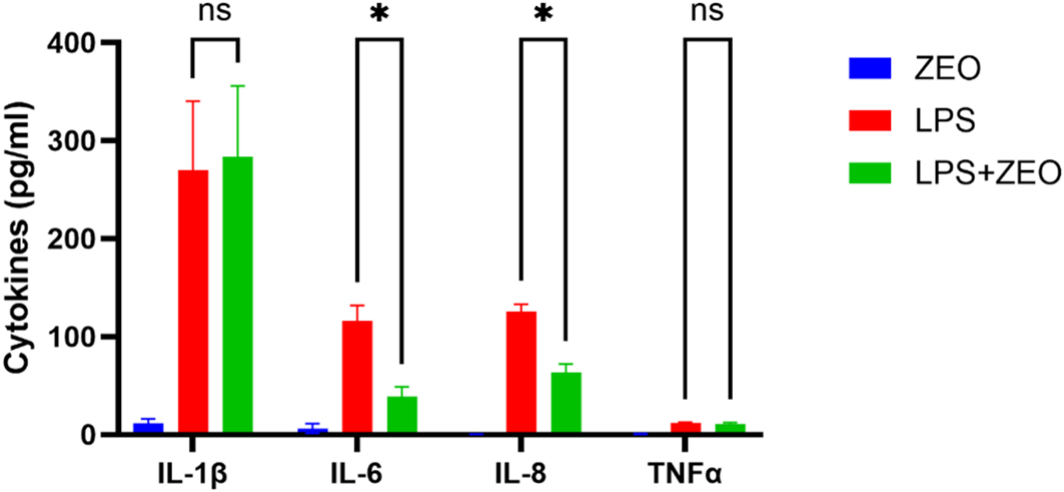
Clinoptilolite zeolite selectively attenuates LPS-induced cytokine production in Caco-2 intestinal epithelial cells. Cytokine concentrations in conditioned media were quantified by ELISA following stimulation with LPS in the presence or absence of clinoptilolite zeolite. Data are presented as mean ± SD. *p < 0.05; ns, not significant.

To further investigate the intestinal axis of clinoptilolite zeolite activity, we examined its effect on LPS-induced cytokine production in Caco-2 epithelial cells (32). Stimulation with LPS significantly increased the secretion of IL-1β, IL-6, IL-8, and TNF-α compared with unstimulated controls (**Figure 5**), confirming activation of epithelial inflammatory signaling following LPS incubation in Caco-2 cells.

Pre-treatment with clinoptilolite zeolite attenuated LPS-induced production of IL-6 and IL-8 (*p* < 0.05), whereas IL-1β and TNF-α levels were not significantly altered (**Figure 5**). Clinoptilolite zeolite alone did not induce cytokine secretion, indicating that the material does not intrinsically activate epithelial inflammatory pathways.

The selective reduction of IL-6 and IL-8 is particularly relevant in the context of intestinal inflammation. IL-6 is a key mediator of epithelial immune signaling and contributes to amplification of inflammatory responses, while IL-8 plays a central role in neutrophil recruitment and mucosal immune activation (32–34). The attenuation of these cytokines suggests that clinoptilolite zeolite reduces specific components of LPS-driven epithelial inflammatory signaling rather than broadly suppressing cytokine production.

Importantly, the absence of an effect on IL-1β and TNF-α indicates that clinoptilolite zeolite does not affect all measured cytokines to the same extent following endotoxin exposure (**Figure 5**) (35, 36). Together with the immune cell data, these findings support the interpretation that clinoptilolite zeolite reduces the availability of LPS to stimulate epithelial and immune cells, resulting in lower measured inflammatory readouts.

### Clinoptilolite zeolite reduces LPS-associated epithelial barrier dysfunction in Caco-2 monolayers

**Figure 6:**
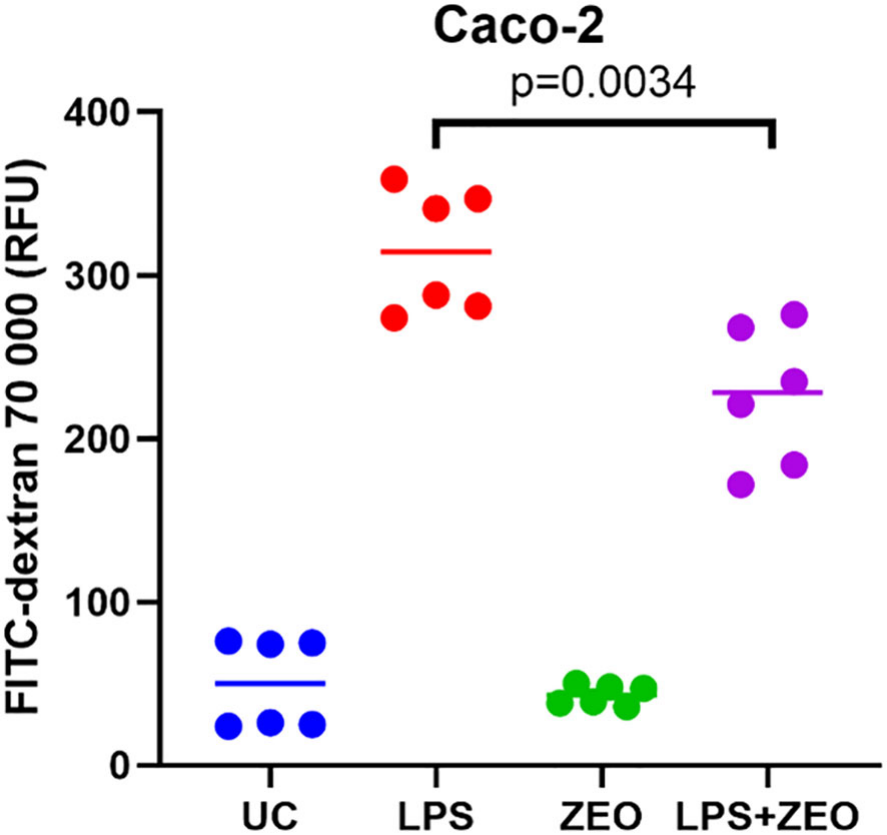
Clinoptilolite zeolite reduces LPS-induced epithelial permeability in Caco-2 monolayers. Caco-2 cells were cultured on 0.4-µm Transwell inserts for 7 days to allow epithelial monolayer formation prior to treatment with lipopolysaccharide (LPS), clinoptilolite zeolite (ZEO), or combined LPS + ZEO conditions for 24 h. Epithelial permeability was subsequently evaluated using FITC-dextran (70 kDa) translocation across the epithelial barrier. Data are presented as relative fluorescence units (RFU) ± SD. Statistical significance was determined using an unpaired Student’s t-test.

To further evaluate whether clinoptilolite zeolite influences downstream inflammatory epithelial responses associated with LPS exposure, epithelial barrier permeability was assessed using a FITC-dextran translocation assay in Caco-2 monolayers cultured on Transwell inserts (**Figure 6**).

Because epithelial barrier disruption represents a functional downstream consequence of inflammatory signaling, this assay was used to determine whether clinoptilolite zeolite modulates LPS-associated epithelial dysfunction beyond cytokine production alone.

Stimulation of Caco-2 monolayers with LPS resulted in a marked increase in FITC-dextran permeability compared with untreated controls, indicating compromised epithelial barrier integrity under inflammatory conditions (**Figure 6**). In contrast, co-treatment with clinoptilolite zeolite significantly reduced LPS-induced FITC-dextran permeability (*p* = 0.0034), suggesting partial preservation of epithelial barrier function during endotoxin exposure. Importantly, treatment with clinoptilolite zeolite alone did not increase epithelial permeability compared with untreated controls (**Figure 6**).

Collectively, these findings suggest that clinoptilolite zeolite reduces downstream functional consequences associated with LPS-induced inflammatory epithelial activation, including epithelial barrier dysfunction. While the precise intracellular signaling mechanisms responsible for these effects remain to be determined, the observed reduction in epithelial permeability is consistent with reduced inflammatory epithelial responses during direct exposure to clinoptilolite zeolite.

### Subchronic oral administration of clinoptilolite zeolite does not induce hematological alterations in rats

**Figure 7:**
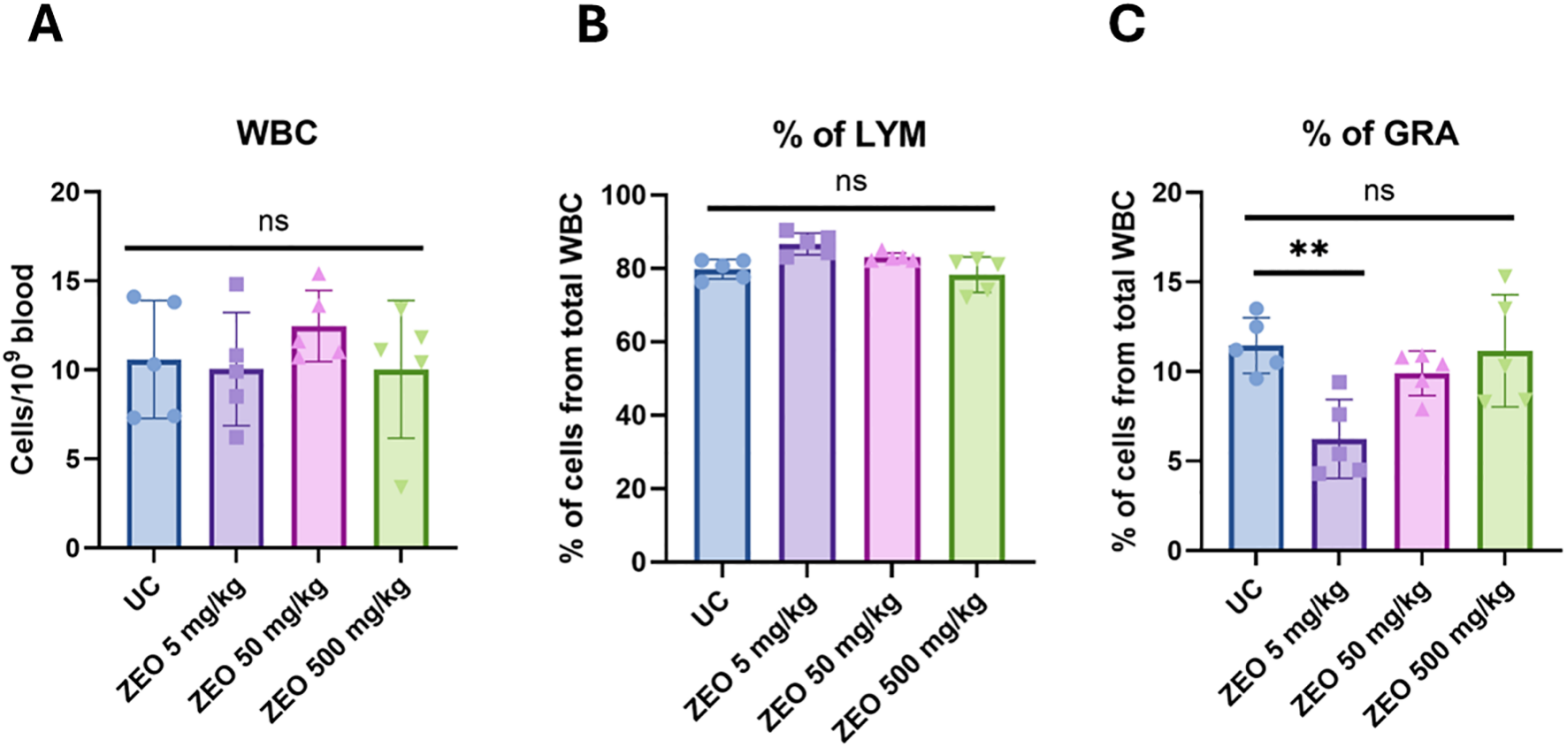
Subchronic oral administration of clinoptilolite zeolite does not induce major alterations in leukocyte parameters in Wistar rats. Animals received daily oral gavage of clinoptilolite zeolite for 29 days. Peripheral blood was collected at the end of the treatment period and analyzed using an automated hematology analyzer to quantify white blood cells **(A)**, %of lymphocytes **(B)**, and % of granulocytes **(C)**. Data are presented as mean ± SD. Statistical analysis was performed using an unpaired Student's t-test. **p < 0.01; ns, not significant.

Following the demonstration that clinoptilolite zeolite attenuates LPS-induced inflammatory responses *in vitro*, we next aimed to evaluate its safety and tolerability *in vivo*. Because the primary objective of the animal study was to assess subchronic safety rather than therapeutic efficacy under inflammatory challenge, animals were not subjected to exogenous LPS administration. Instead, clinically healthy Wistar rats received daily oral doses of clinoptilolite zeolite (5, 50, or 500 mg/kg) for 29 days to determine whether repeated exposure induced hematological changes (**Figure 7**).

Total white blood cell (WBC) counts were not significantly different between control and clinoptilolite zeolite-treated groups (**Figure 7**), indicating that clinoptilolite zeolite administration did not induce leukocytosis or leukopenia (37, 38). Similarly, the relative proportion of lymphocytes (% LYM) remained comparable across all groups (**Figure 7B**). Although a statistically significant reduction in granulocyte percentage (% GRA) was observed at the lowest dose (5 mg/kg), this effect was not dose-dependent and remained within established physiological reference ranges. Importantly, no consistent dose-dependent pattern in granulocyte distribution was detected across treatment groups (**Figure 7C**) (39).

**Figure 8:**
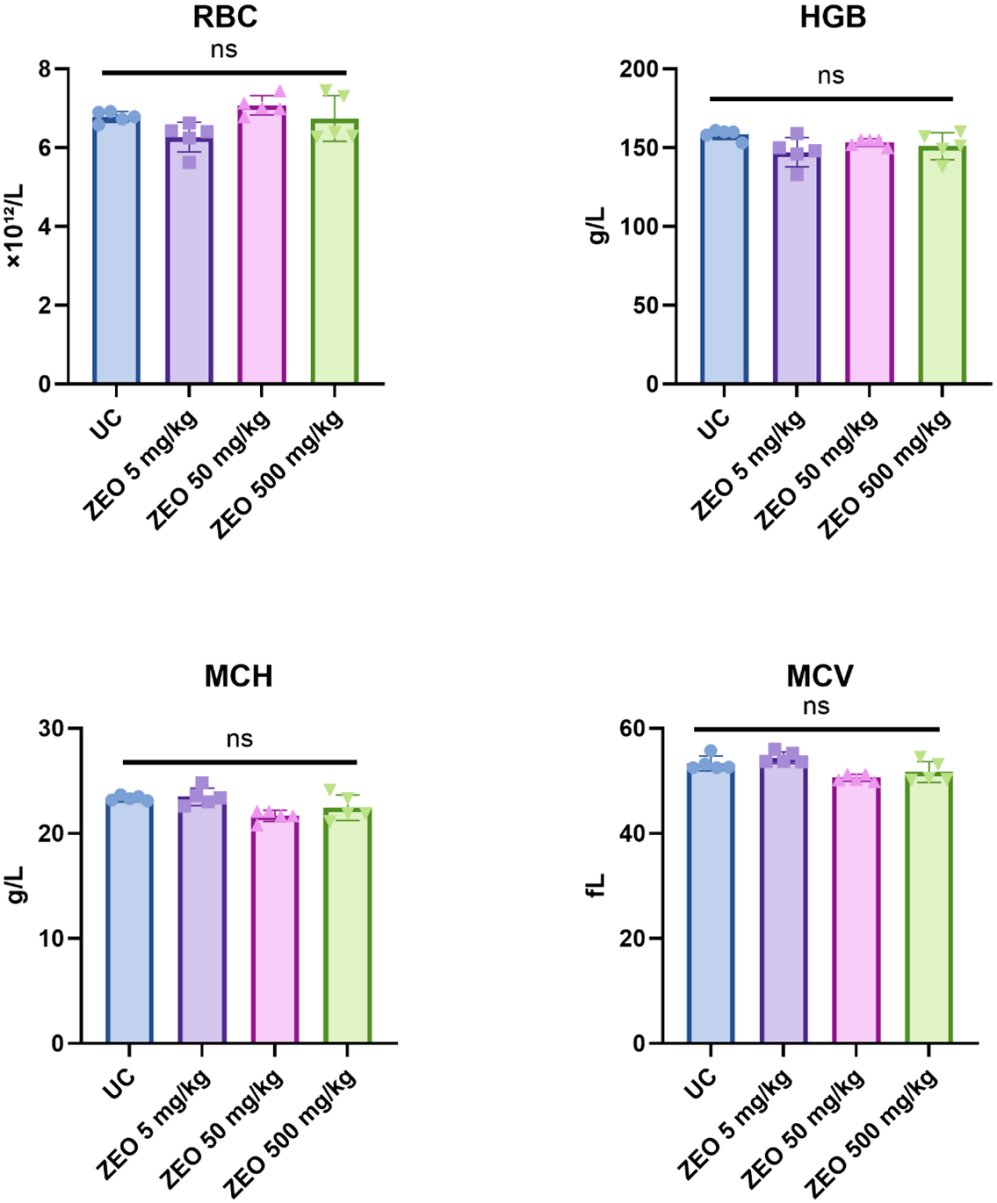
Subchronic oral administration of clinoptilolite zeolite does not affect erythrocyte markers in Wistar rats. Animals received daily oral gavage of clinoptilolite zeolite for 29 days. Peripheral blood samples were collected at the end of the treatment period and analyzed using an automated hematology analyzer. Red blood cell count (RBC), hemoglobin concentration (HGB), mean corpuscular hemoglobin (MCH), and mean corpuscular volume (MCV) were evaluated. Data are presented as mean ± SD. Statistical analysis was performed using an unpaired Student’s t-test; ns, not significant.

Similarly, hemoglobin concentration (HGB), mean corpuscular hemoglobin (MCH), and mean corpuscular volume (MCV) showed no significant differences between groups. No dose-dependent trends were observed, and all measured values remained within established physiological reference ranges for Wistar rats (**Figure 8**). The absence of changes in RBC indicates that clinoptilolite zeolite administration did not induce anemia, erythrocyte structural changes, or disturbances in hemoglobin synthesis. In particular, stable MCV and MCH values suggest preserved erythrocyte morphology and hemoglobin content, further supporting hematological stability during daily oral administration over the 29-day exposure period (40, 41).

**Figure 9:**
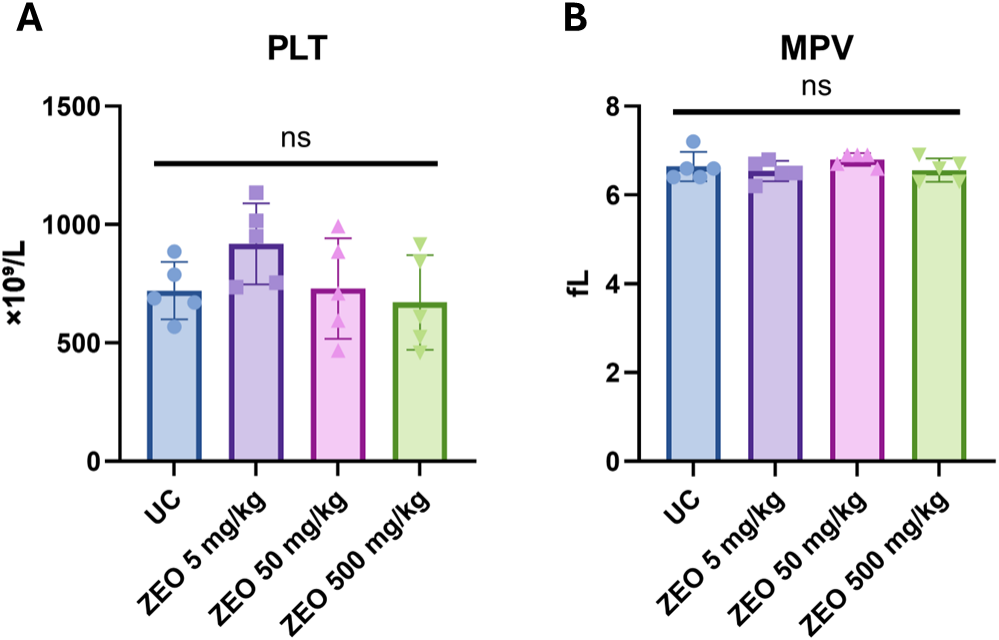
Subchronic oral administration of clinoptilolite zeolite does not alter platelet parameters in Wistar rats. Animals received daily oral gavage of clinoptilolite zeolite (ZEO) for 29 days. Peripheral blood samples were analyzed using an automated hematology analyzer. **(A)** Platelet count (PLT). **(B)** Mean platelet volume (MPV). Data are presented as mean ± SD. Statistical analysis was performed using an unpaired Student’s *t*-test; ns, not significant.

As shown in **Figure 9**, platelet counts (PLT) were comparable between control and clinoptilolite zeolite-treated groups across all tested doses (5, 50, and 500 mg/kg). No statistically significant differences were observed, and no dose-dependent trends were detected.

Collectively, hematological evaluation revealed no clinically relevant alterations following subchronic oral administration of clinoptilolite zeolite. Total leukocyte counts and differential distribution remained largely unchanged, with no evidence of leukocytosis, leukopenia, or dose-dependent immune cell redistribution. Erythrocyte parameters, including RBC count, hemoglobin concentration, MCV, and MCH, were comparable to control animals, indicating preserved erythropoiesis and absence of anemia or red cell morphological disturbances. Similarly, platelet count and mean platelet volume were unaffected across all tested doses. Together, these findings demonstrate that repeated oral exposure to clinoptilolite zeolite at doses up to 500 mg/kg does not induce hematological toxicity and support its systemic hematological tolerability under the conditions evaluated.

### Histological evaluation reveals no treatment-related tissue alterations following subchronic clinoptilolite zeolite administration

**Figure 10:**
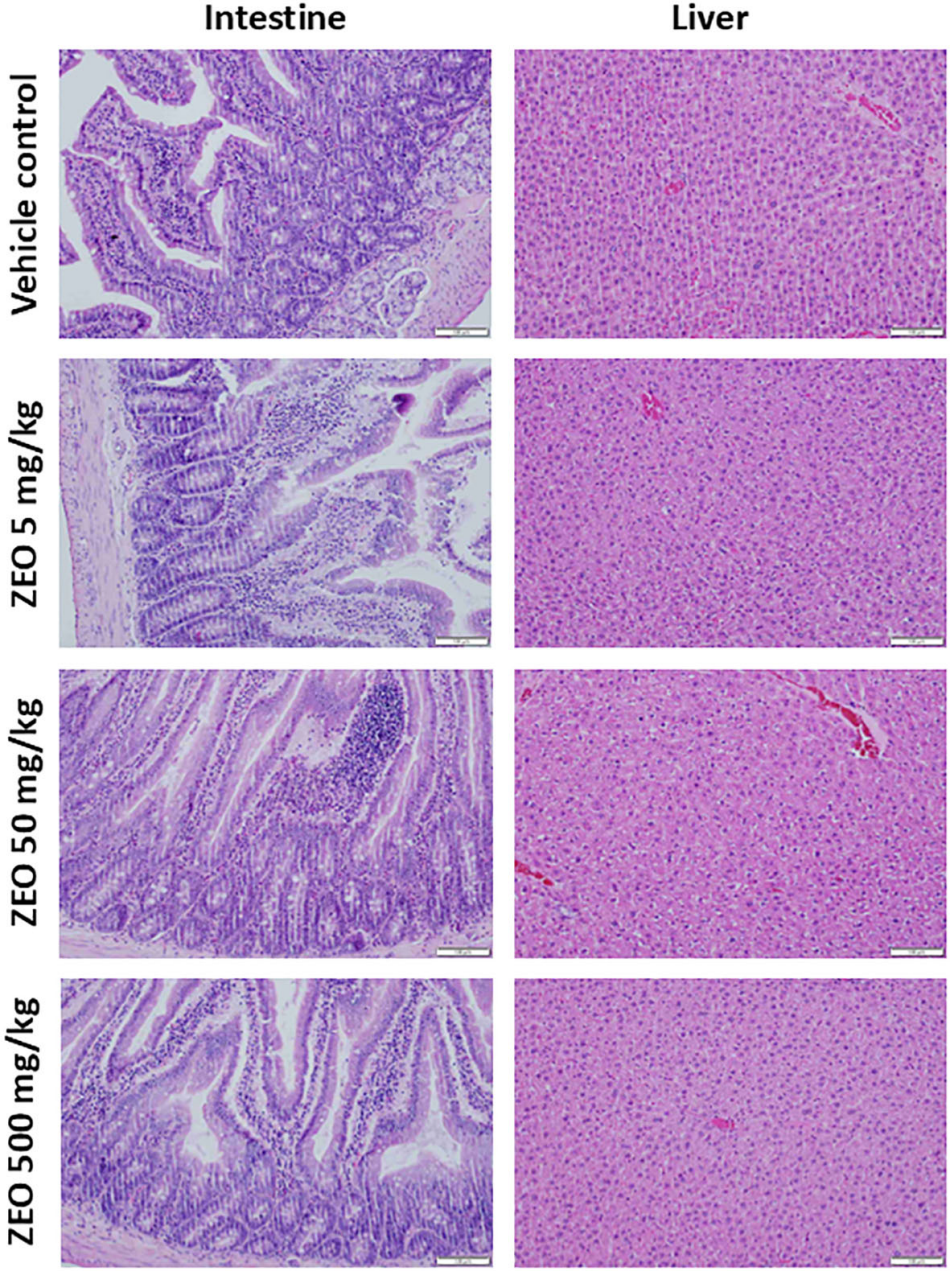
Representative histological sections of intestine and liver following subchronic oral administration of clinoptilolite zeolite. Animals received daily oral gavage of clinoptilolite zeolite for 29 days. Intestinal and hepatic tissues were collected at the end of the treatment period, fixed in formalin, embedded in paraffin, sectioned, and stained with hematoxylin and eosin (H&E). Representative images are shown for vehicle control and clinoptilolite zeolite-treated groups. Scale bars: 100 µm.

**Figure 11:**
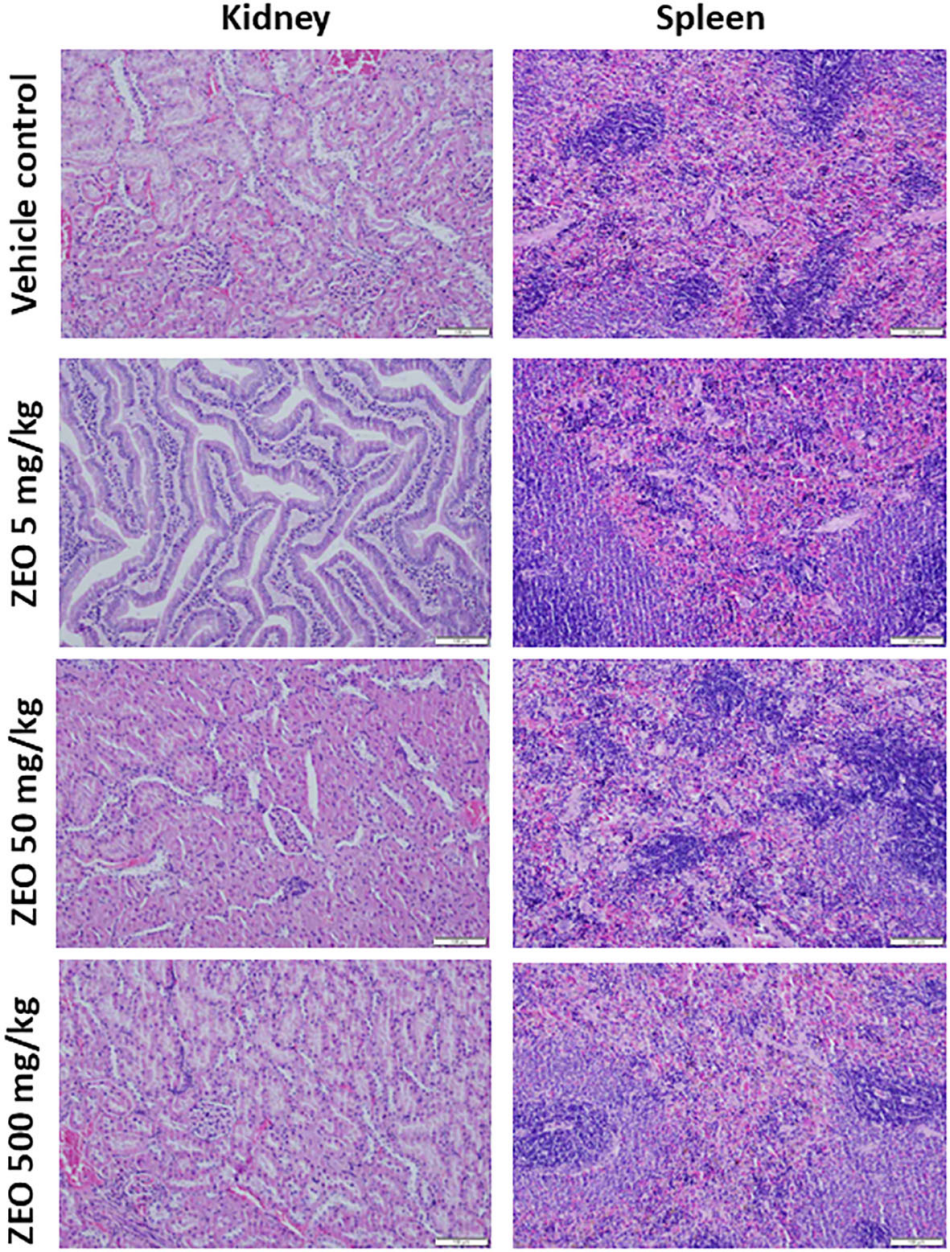
Representative histological sections of the kidney and spleen following subchronic oral administration of clinoptilolite zeolite. Animals received daily oral gavage of clinoptilolite zeolite for 29 days. Kidney and spleen tissues were collected at the end of the treatment period, fixed in formalin, embedded in paraffin, sectioned, and stained with hematoxylin and eosin (H&E). Representative images from vehicle control and clinoptilolite zeolite-treated groups are shown. Scale bars: 100 µm.

**Figure 12:**
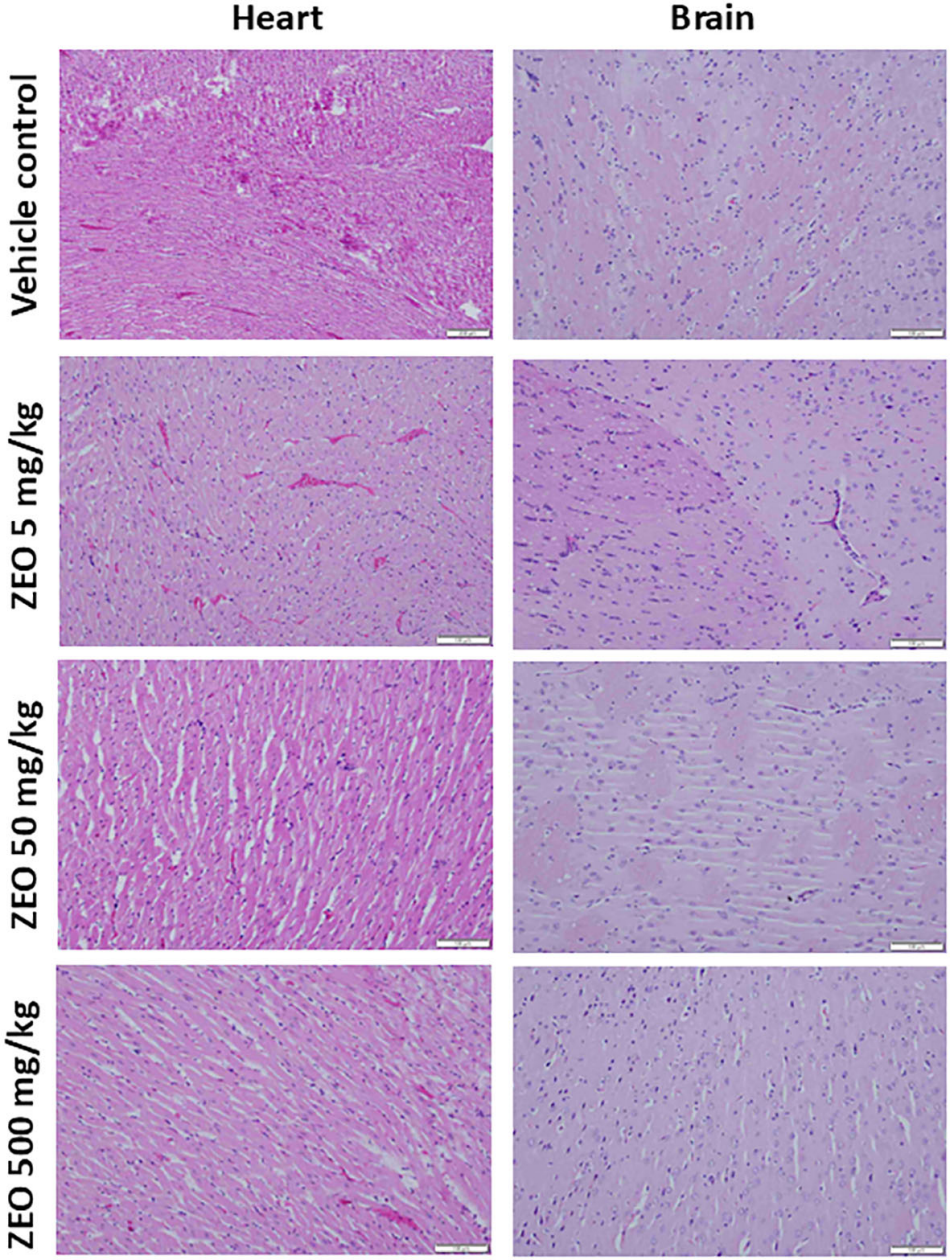
Representative histological sections of the heart and brain following subchronic oral administration of clinoptilolite zeolite. Animals received daily oral gavage of clinoptilolite zeolite for 29 days. Heart and brain tissues were collected at the end of the treatment period, fixed in formalin, embedded in paraffin, sectioned, and stained with hematoxylin and eosin (H&E). Representative images from vehicle control and clinoptilolite zeolite-treated groups are shown. Scale bars: 100 *µ*m.

To further assess systemic tolerability, histological evaluation of major organs was performed following subchronic oral administration of clinoptilolite zeolite. Hematoxylin and eosin (H&E)-stained sections of intestine and liver (**Figure 10**), kidney and spleen (**Figure 11**), and heart and brain (**Figure 12**) were examined by a pathologist blinded to the experimental design.

Across all tested doses (5, 50, and 500 mg/kg), tissue architecture remained preserved and comparable to vehicle-treated controls. Intestinal sections demonstrated intact villous structure, normal epithelial integrity, and no evidence of inflammatory cell infiltration or mucosal damage (**Figure 10**).

Liver tissue displayed preserved hepatocellular organization with no signs of necrosis, steatosis, or inflammatory infiltration (**Figure 10**). Renal sections showed normal glomerular and tubular morphology without evidence of degeneration or interstitial inflammation. Splenic architecture, including white and red pulp organization, was maintained across all groups (**Figure 11**).

Similarly, cardiac tissue exhibited preserved myocardial fiber alignment without inflammatory changes or structural abnormalities. Brain sections showed normal neuronal morphology and no detectable signs of edema, necrosis, or inflammatory infiltration (**Figure 12**).

Importantly, no dose-dependent histopathological alterations were observed in any examined organ.

Collectively, the *in vivo* findings demonstrate that subchronic oral administration of clinoptilolite zeolite at doses up to 500 mg/kg is well tolerated in Wistar rats (42). Comprehensive hematological evaluation revealed no clinically relevant alterations in leukocyte distribution, erythrocyte indices, or platelet parameters. Together, these results indicate that repeated oral exposure to clinoptilolite zeolite does not induce detectable systemic toxicity under the experimental conditions employed and support its favorable tolerability profile *in vivo*.

## Conclusions

In this study, we demonstrate that natural clinoptilolite zeolite attenuates LPS-induced inflammatory responses *in vitro* and exhibits a favorable tolerability profile following subchronic oral administration in rats. Clinoptilolite zeolite reduced NF-κB activation and nitric oxide production in innate immune cell models and selectively decreased pro-inflammatory cytokine production in intestinal epithelial cells. Functional pre-incubation experiments further support the notion that clinoptilolite zeolite reduces the biological activity of LPS, likely by decreasing endotoxin bioavailability. Importantly, repeated oral administration at doses up to 500 mg/kg did not induce hematological or histopathological changes in major organs, indicating systemic tolerability under the tested conditions (42, 43).

Collectively, these findings provide *in vitro* and *in vivo* proof-of-concept evidence supporting the immunomodulatory potential and safety of clinoptilolite zeolite as a functional food additive material. However, several limitations should be acknowledged. The mechanistic basis of LPS interaction was assessed functionally rather than through direct biochemical quantification, and the *in vivo* study was designed to evaluate safety rather than efficacy under inflammatory challenge conditions. The adsorption-mediated mechanism proposed in this study is unlikely to be specific to endotoxin alone. Due to its ion-exchange capacity and porous aluminosilicate framework, clinoptilolite may also interact with biologically relevant ions, micronutrients, or orally administered compounds. Although no hematological evidence of anemia or erythrocyte abnormalities was observed following repeated oral administration, the present study did not directly evaluate iron metabolism or trace element homeostasis. Future investigations should therefore assess the long-term effects of clinoptilolite exposure on micronutrient absorption, including iron, zinc, copper, and selenium bioavailability, particularly under chronic dietary exposure conditions. Additionally, the present study did not evaluate the stability of the proposed clinoptilolite–LPS interaction under physiologically relevant gastrointestinal conditions. Because adsorption processes may be influenced by pH, ionic strength, and competing biomolecules, it remains unclear whether endotoxin association with clinoptilolite is stable throughout gastric and intestinal transit or whether partial desorption may occur. Future studies using simulated gastric and intestinal environments will therefore be important to characterize adsorption/desorption kinetics and residual endotoxin bioavailability under physiologically relevant conditions.

Importantly, all *in vitro* stimulation experiments were conducted in serum-containing culture medium. Therefore, clinoptilolite-mediated reduction of LPS bioactivity occurred in the presence of serum proteins and other soluble biomolecules that may also interact with the zeolite surface. These findings suggest that the observed effects likely reflect complex adsorption dynamics rather than selective endotoxin binding alone. Future studies should further characterize interactions between clinoptilolite and serum-derived proteins, micronutrients, and other biologically relevant soluble factors.

Therefore, further studies are warranted to elucidate the precise molecular mechanisms underlying endotoxin neutralization, assess long-term safety under chronic exposure conditions, and evaluate functional efficacy in disease-relevant *in vivo* models. Such investigations will be essential to fully define the translational potential of clinoptilolite zeolite as a dietary material capable of modulating inflammatory responses at the intestinal interface.

## Data Availability

The original contributions presented in the study are included in the article/**Supplementary Material**. Further inquiries can be directed to the corresponding author.
