# Supplementary material for: Adsorption-mediated modulation of lipopolysaccharide bioactivity by clinoptilolite zeolite with in vitro immunomodulatory effects and in vivo safety evaluation
Source: Front Immunol. 2026 Jul 30;17:1818740. doi: 10.3389/fimmu.2026.1818740 (PMC13468899; doi:10.3389/fimmu.2026.1818740)
Supplement: Supplementary file 1 [file Table1.docx]

**Adsorption-Mediated Modulation of Microbial Endotoxin Bioactivity by Clinoptilolite Zeolite with In Vitro Immunomodulatory Effects and In Vivo Safety Evaluation**

**Povilas Kavaliauskas *^1,2,3^, Rolandas Stankevicius ^1^, Julius Rakickas ^4^, Alius Pockevicius ^5^, Jonas Simkevicius ^6^, and Ramune Grigaleviciute ^1,2^.**

**SUPLEMENTARY FILES**

1. Department of Animal Nutrition, Lithuanian University of Health Sciences, Tilzes Str. 18, LT-47181 Kaunas, Lithuania.
2. Biological Research Center, Lithuanian University of Health Sciences, Tilzes Str. 18/7, LT-47181 Kaunas, Lithuania.
3. Institute of Infectious Diseases and Pathogenic Microbiology, Birstono Str. 38A, LT-59116 Prienai, Lithuania.
4. Public Institution Šilutė Primary Health Care Center, Šilutė, Lithuania
5. Department of Veterinary Pathobiology, Lithuanian University of Health Sciences, Kaunas, Lithuania.
6. UAB Nutrex.

* to whom correspondence should be addressed: povilas.kavaliauskas@lsmu.lt

Figure S1. The chemical composition of zeolite provided by manufacturer.
