# Supplementary material for: Adsorption-mediated modulation of lipopolysaccharide bioactivity by clinoptilolite zeolite with in vitro immunomodulatory effects and in vivo safety evaluation
Source: Front Immunol. 2026 Jul 30;17:1818740. doi: 10.3389/fimmu.2026.1818740 (PMC13468899; doi:10.3389/fimmu.2026.1818740)
Supplement: Supplementary file 2 [file Table2.docx]

**Adsorption-Mediated Modulation of Microbial Endotoxin Bioactivity by Clinoptilolite Zeolite with In Vitro Immunomodulatory Effects and In Vivo Safety Evaluation**

**Povilas Kavaliauskas *^1,2,3^, Rolandas Stankevicius ^1^, Julius Rakickas ^4^, Alius Pockevicius ^5^, Jonas Simkevicius ^6^, and Ramune Grigaleviciute ^1,2^.**

**SUPLEMENTARY FILES**

1. Department of Animal Nutrition, Lithuanian University of Health Sciences, Tilzes Str. 18, LT-47181 Kaunas, Lithuania.
2. Biological Research Center, Lithuanian University of Health Sciences, Tilzes Str. 18/7, LT-47181 Kaunas, Lithuania.
3. Institute of Infectious Diseases and Pathogenic Microbiology, Birstono Str. 38A, LT-59116 Prienai, Lithuania.
4. Public Institution Šilutė Primary Health Care Center, Šilutė, Lithuania
5. Department of Veterinary Pathobiology, Lithuanian University of Health Sciences, Kaunas, Lithuania.
6. UAB Nutrex.

* to whom correspondence should be addressed: povilas.kavaliauskas@lsmu.lt

Table S1. The chemical composition of zeolite provided by manufacturer.

**Figure S1.** Clinoptilolite zeolite does not induce substantial membrane-associated cytotoxicity under experimental conditions used for inflammatory stimulation assays. Lactate dehydrogenase (LDH) release was quantified in conditioned media collected from THP-1 Dual cells (A), RAW264.7 macrophages (B), and Caco-2 epithelial cells (C) following treatment with clinoptilolite zeolite (ZEO), lipopolysaccharide (LPS), phorbol 12-myristate 13-acetate (PMA), or corresponding combination treatments. Data are presented as mean ± SD. Statistical significance was determined using unpaired Student’s t-test. ns, not significant.

**Figure S2.** Schematic representation of the clinoptilolite zeolite pre-incubation and removal assay. THP-1 Dual NF-κB reporter cells were exposed to clinoptilolite zeolite (ZEO) placed in 0.4 µm transwell inserts for 1 h. Following incubation, transwell inserts containing clinoptilolite zeolite were removed, and cells were washed prior to lipopolysaccharide (LPS) stimulation. This experimental design was used to determine whether prior exposure to clinoptilolite zeolite alters subsequent cellular responsiveness to LPS independently of direct extracellular LPS sequestration.
